# Supplementary material for: Pathological mechanisms and candidate therapeutic approaches in the hearing loss of mice carrying human MIR96 mutations
Source: Genome Med. 2024 Oct 21;16:121. doi: 10.1186/s13073-024-01394-5 (PMC11492784; doi:10.1186/s13073-024-01394-5)
Supplement: Supplementary file 3 — Additional file 3: Supplementary Figures S1–S4. [file 13073_2024_1394_MOESM3_ESM.docx]

**
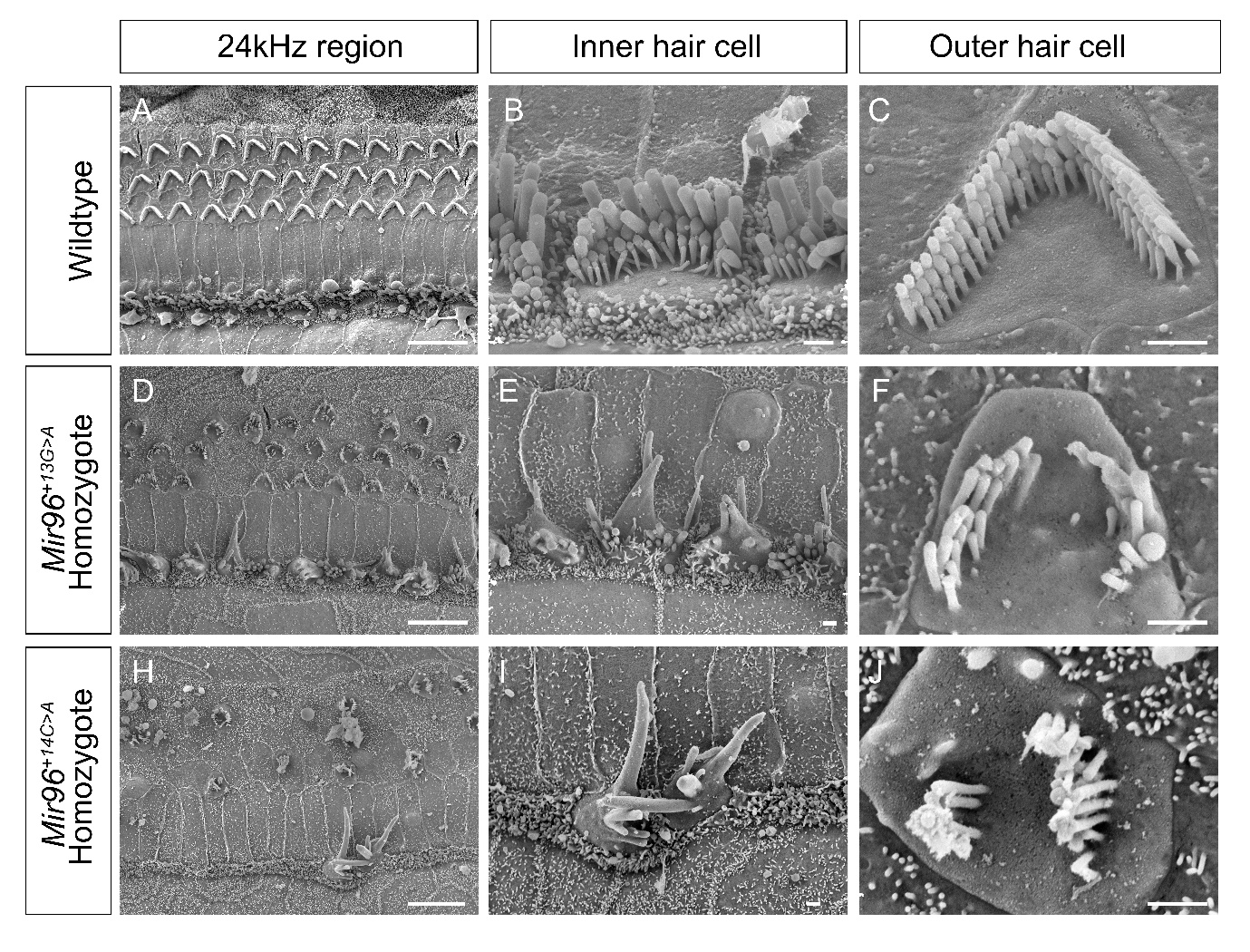
**

**Figure S1. Scanning electron micrographs of *Mir96^+13G>A^* (D-F) and *Mir96^+14C>A^* (H-J) homozygous mutant mice at 4 weeks old.** Representative examples of wildtype (A-C), *Mir96^+13G>A^* (D-F) and *Mir96^+14C>A^* (H-J) mice are shown. All images correspond to the 24 kHz best frequency region. For each panel, the left column (A, D, H) shows a zoomed-out image with inner and outer hair cell rows. The middle column (B, E, I) shows an inner hair cell close up, and the right column (C, F, J) shows an outer hair cell close up. *Mir96^+13G>A^* mice: wildtype (n=4), heterozygote (n=3), homozygote (n=3). *Mir96^+14C>A^* mice: wildtype (n=3), heterozygote (n=6), homozygote (n=3). Scale bar on left hand panels = 10 µm; scale bar for single hair cells = 1 µm.

**
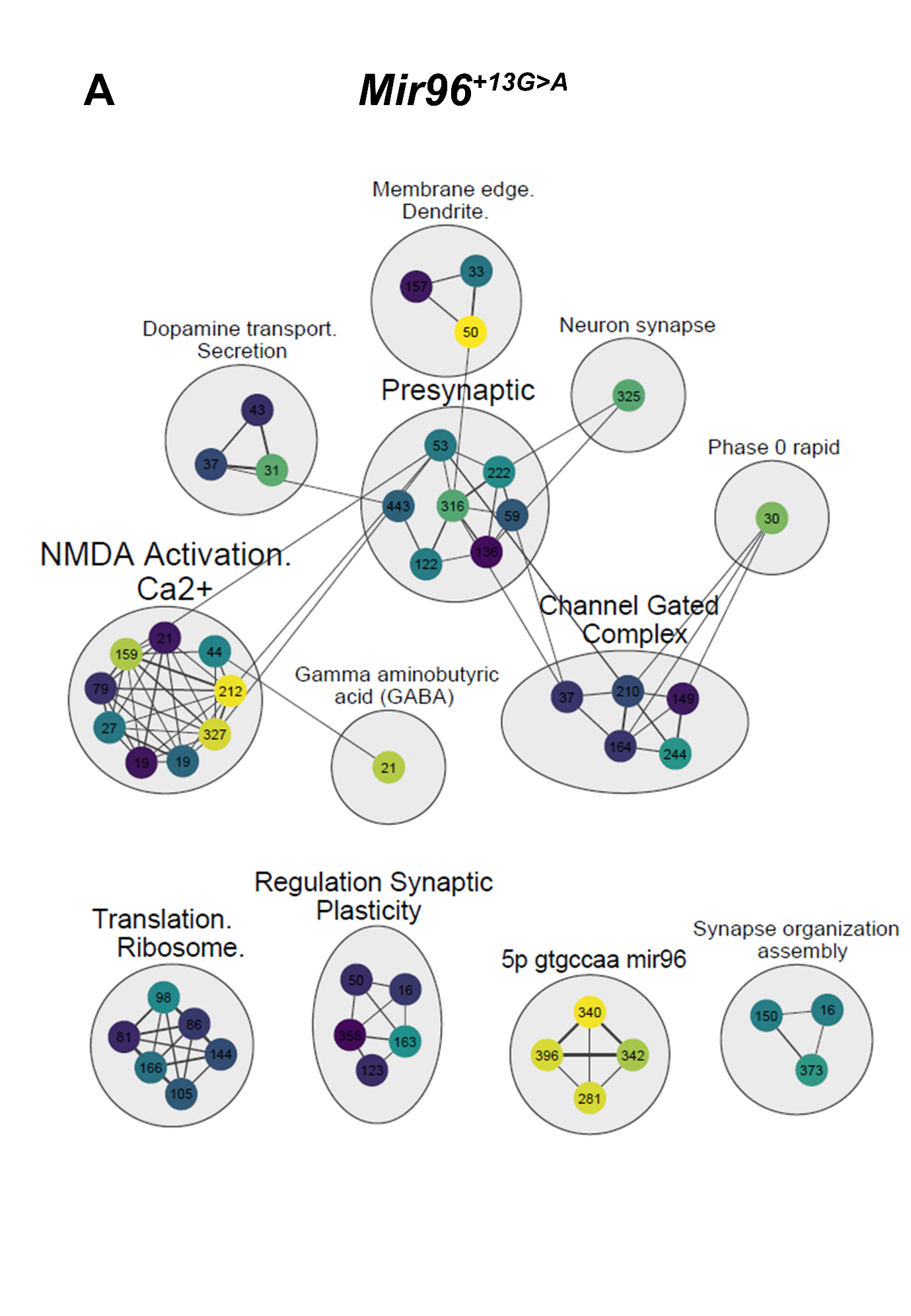
**

**
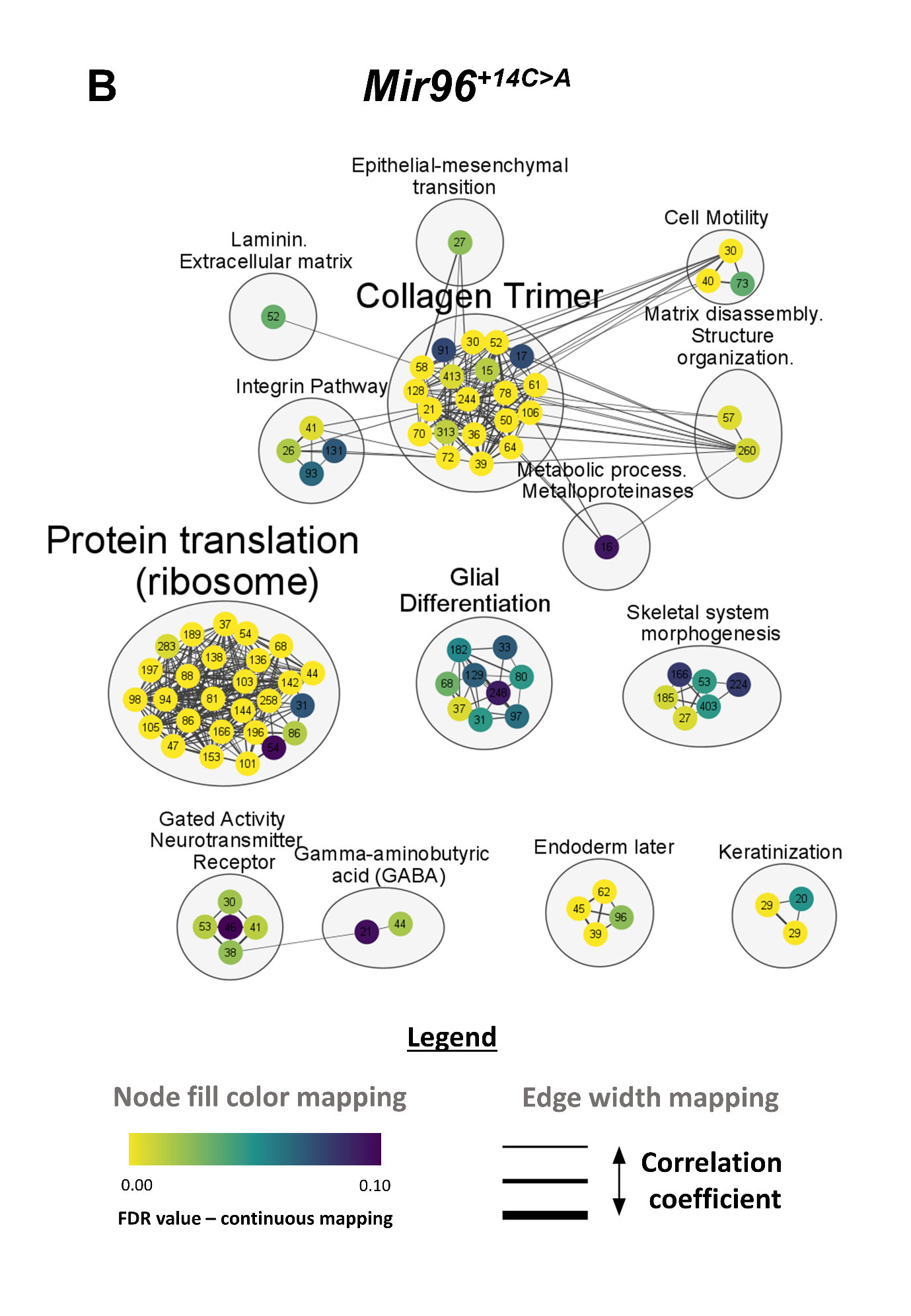
**

**Figure S2. Enrichment map visualization in Cytoscape.** A) Enrichment map based on GSEA analysis of the *Mir96^+13G>A^* mutant transcriptome. B) Enrichment map based on GSEA analysis of the *Mir96^+14C>A^* mutant transcriptome. Each map contains gene sets (nodes) interconnected by the similarity between them (edges). The colour of the nodes is based on the FDR, with a continuous colour scheme from yellow (FDR=0) to purple (FDR=0.1). In other words, the nodes in yellow correspond to gene sets with a high number of genes shared with our list of DEG. The more purple the node, the less similarity between the gene set and our list of DEGs. The number of genes in each gene set is indicated inside each node. The thickness of the edges represents the similarity coefficient: the more similar two gene sets the thicker the edge that connects them. The nodes with a degree of 1 or 0 that were not connected to the network were removed. To define major biological clusters represented in these gene sets we used the tool AutoAnnotate which automatically defines clusters within the network. AutoAnnotate first clusters the network using the clusterMaker2 application in Cytoscape and draws a circle around each cluster. Then, an annotation associated with the three words more represented in the cluster is added using the WordCloud app. These annotations have been manually curated to ease data interpretation. The size of the cluster label reflects the number of nodes of that cluster.

**
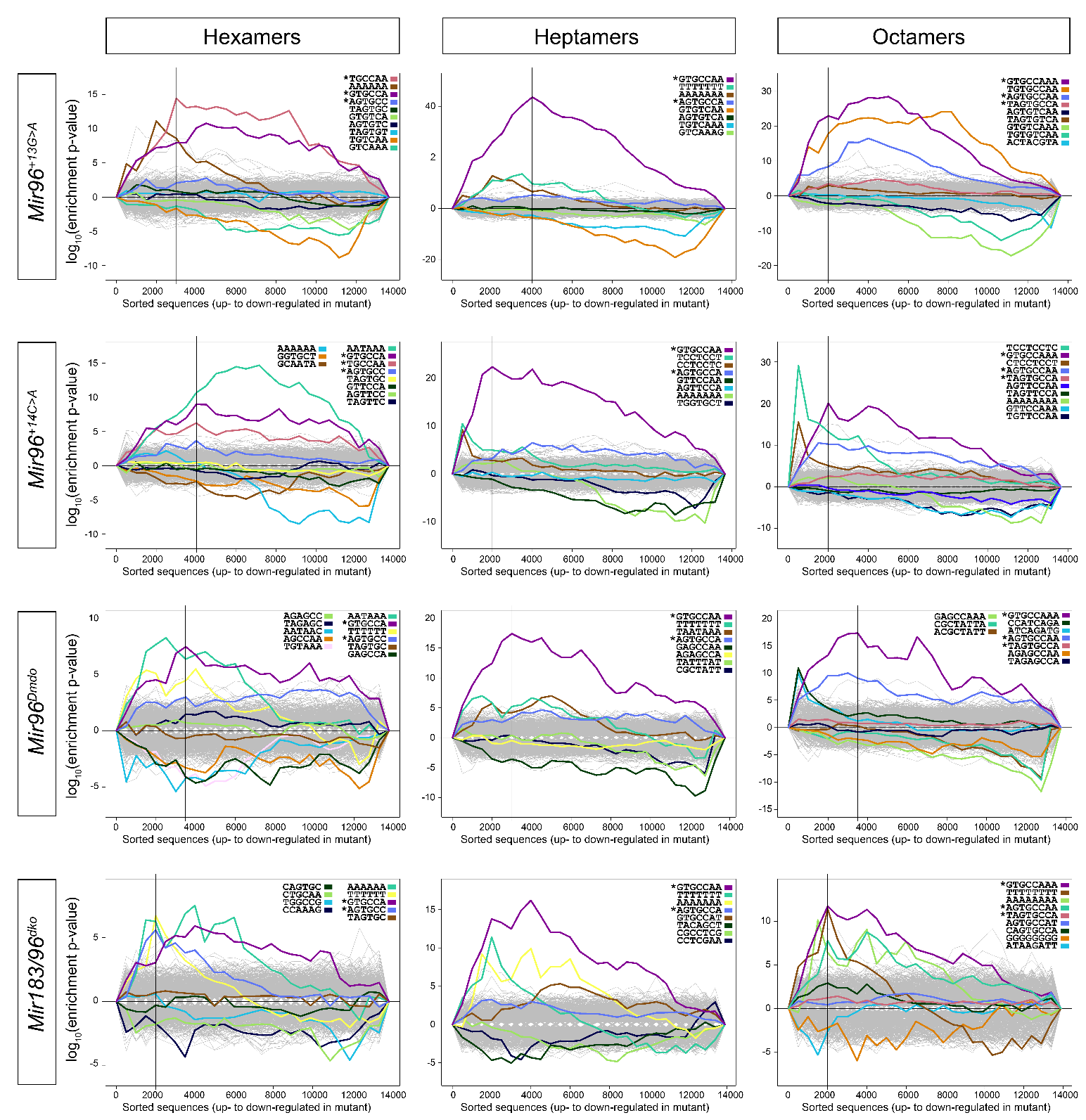
Figure S3. Sylamer plots of hexamers, heptamers and octamers in the misregulated transcriptomes of four *Mir96* mutants.** For *Mir183/96^dko^* and *Mir96^Dmdo^*, the RNAseq data from [12] and the microarray data from [4] were used respectively, and replotted with 3’UTR data from GRCm39. Starred words indicate wildtype seed region matches. The vertical line on each plot shows the location of the peak enrichment of the wildtype seed(s), which was determined to be the first peak of a word corresponding to a wildtype seed region. Candidate targets are the genes to the left of this line (so the most upregulated genes) which have a wildtype seed region match in their 3’UTR.

**
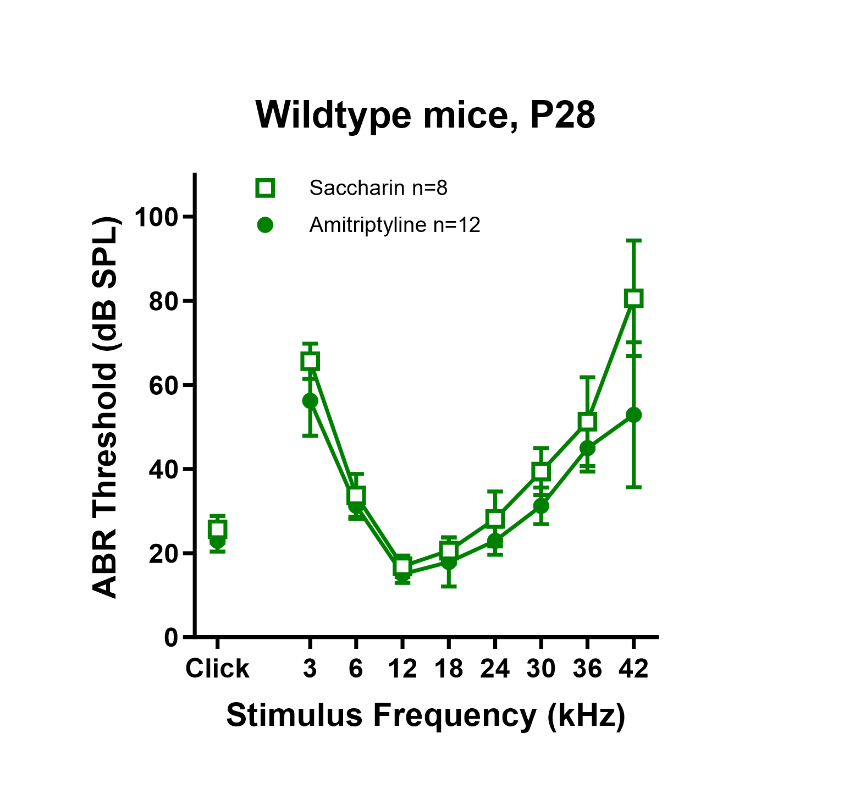
**

**Figure S4. Testing amitriptyline on C57BL/6N wildtype mice.** No difference in thresholds was observed between wildtype mice given 200µg/ml amitriptyline and 2% saccharin in their water and wildtype mice given just 2% saccharin at 4 weeks old.
